# Statistical and machine learning approaches for identifying biomarker associations in respiratory diseases in a population-specific region

**DOI:** 10.3389/frai.2025.1682774

**Published:** 2025-11-27

**Authors:** Meshari Alazmi, Amer AlGhadhban, Abdulaziz Almalaq, Kamaleldin B. Said, Yazeed Faden

**Affiliations:** 1 College of Computer Science and Engineering, University of Hail, Hail, Saudi Arabia; 2 Medical and Diagnostic Research Center, University of Hail, Hail, Saudi Arabia; 3 College of Engineering, University of Hail, Hail, Saudi Arabia; 4 Department of Pathology, College of Medicine, University of Hail, Hail, Saudi Arabia; 5 Faculty of Computing and Information Technology, King Abdulaziz University, Rabigh, Saudi Arabia

**Keywords:** biomarkers, respiratory diseases, COVID-19, machine learning, decision tree, pulmonology, non-invasive diagnostics, clinical data analysis

## Abstract

The growing interest in utilizing clinical blood biomarkers for non-invasive diagnostics has transformed the approach to early detection and prognosis of respiratory diseases. Biomarker-driven diagnostics offer cost-effective, rapid, and scalable alternatives to traditional imaging and clinical assessments. In this study, we conducted a retrospective analysis of 913 patients from a local respiratory clinic in Hail region, evaluating the diagnostic relevance of 15 blood biomarkers across four respiratory conditions: COVID-19, pneumonia, asthma, and other complications. Through data-driven analysis, statistical correlation assessments, and machine learning classification models (decision tree classifiers), we identified significant biomarker interactions that contributed to disease differentiation. Notably, CRP and HGB demonstrated a strong negative correlation (−55%), supporting the well-established role of systemic inflammation in anemia of chronic disease. Additionally, Ferritin and LDH exhibited a positive correlation (+50%), indicating metabolic stress and cellular injury in severe respiratory illnesses. Other significant correlations included Creatinine and ESR being negatively associated with RBC, while GGT and ALT were positively correlated (+49%). Additionally, bilirubin and HGB were positively correlated (+49%), collectively reflecting systemic inflammatory and metabolic responses associated with respiratory pathology. The machine learning model demonstrated high predictive accuracy, with the following performance metrics: COVID-19: Precision (0.94), Recall (0.96), F1-score (0.95). Pneumonia: Precision (0.97), Recall (0.71), F1-score (0.85). Asthma: Precision (1.00), Recall (0.95), F1-score (0.97). Other Complications: Precision (0.88), Recall (0.90), F1-score (0.90). These findings validate the diagnostic potential of biomarker panels in respiratory disease classification, offering a novel approach to integrating statistical and computational modeling for clinical decision-making. By leveraging biomarker relationships and machine learning algorithms, this study contributes to the development of personalized, non-invasive, and cost-effective diagnostic tools for respiratory diseases, ultimately improving patient outcomes and healthcare efficiency.

## 1 Introduction

The early and accurate diagnosis of respiratory diseases holds the potential to enhance patient outcomes while simultaneously reducing healthcare costs (Larsson et al., 2019). Recently, there has been a growing interest in clinical blood biomarkers for non-invasive diagnosis and prognosis of various lung diseases (Liu et al., 2021). Biomarkers such as C-reactive protein (CRP), procalcitonin, and club cell protein 16 (CC16) have been investigated for their association with inflammatory lung diseases like COPD (Rosenberg and Kalhan, 2012). Elevated levels of fibrinogen and white blood cell counts have been reported in COPD patients, indicating their potential as prognostic markers (Thomsen et al., 2012).

The recent development in lung cancer diagnosis has shown progress through the investigation of blood-based biomarkers, such as circulating tumor DNA (ctDNA), microRNAs (miRNAs), and certain proteins that display promising potential for early detection (Saman et al., 2022). In a notable study by Smith et al., a combination of multiple biomarkers, including carcinoembryonic antigen (CEA) and cytokeratin 19 fragment (CYFRA 21-1), demonstrated the potential to enhance the predictive accuracy for lung cancer diagnosis (Okamura et al., 2013). Similarly, pulmonary fibrosis has been associated with increased levels of matrix metalloproteinases (MMPs) and Krebs von den Lungen-6 (KL-6) in the blood (Hamai et al., 2016). The use of advanced techniques like mass spectrometry and next-generation sequencing has facilitated the identification and validation of novel biomarkers with high sensitivity and specificity (Wheelock et al., 2013).

Nonetheless, challenges remain in standardizing these blood biomarker tests; factors such as age, sex, and comorbid conditions can influence their levels (Chang and Wu, 2022). The future trajectory of this field encompasses the integration of clinical, imaging, and multi-omics data to develop comprehensive models for lung disease prediction (Blutt et al., 2023).

Respiratory diseases encompass a wide range of pathologies, from chronic conditions like COPD and asthma to acute and severe diseases such as lung cancer. The search for non-invasive diagnostic and prognostic markers has led researchers to examine various clinical blood biomarkers. For instance, several studies have highlighted the importance of CRP as a potential marker for COPD and its exacerbations (Ridker, 2007). Elevated CRP levels in the blood indicate inflammation and have been associated with increased risk and severity of COPD (Mannino et al., 2012). The procalcitonin biomarker, mainly tied to bacterial infections, has been investigated for its potential in distinguishing bacterial from viral pneumonia—an insight which can help in appropriate antibiotic administration (Christ-Crain and Müller, 2005). Elevated levels of the Cytokeratin-19 fragment (CYFRA 21-1) have been identified in patients with non-small cell lung cancer (NSCLC), suggesting its potential role as a diagnostic or prognostic marker (Pujol et al., 1996). Emerging studies focusing on lung cancer emphasize the potential of circulating tumor DNA (ctDNA) as a minimally invasive technique for detecting mutations, monitoring treatment response, and potentially predicting disease recurrence (Wan et al., 2017). Club cell secretory protein (CC16) levels have been explored for their relation with lung function decline and COPD risk. Lower serum CC16 levels have been linked to an elevated risk of COPD and rapid lung function deterioration (Guerra et al., 2015). An accumulating list of evidence suggests that the Neutrophil-to-lymphocyte ratio (NLR) could be a promising prognostic marker for various lung diseases, including NSCLC. Elevated NLR is linked with poorer outcomes and decreased survival rates in NSCLC patients (Cedrés et al., 2012). Additionally, soluble ST2 (sST2) levels have demonstrated associations with idiopathic pulmonary fibrosis (IPF) severity and prognosis. Elevated sST2 levels may indicate a higher risk of disease progression and mortality (Yu et al., 2022).

Correlations between various blood markers and respiratory diseases have been extensively explored in clinical medicine. Many of these markers offer insights into the inflammatory, metabolic, and structural status of the body, relevant to respiratory conditions. Elevated C-reactive protein (CRP) levels typically indicate inflammation, with conditions like pneumonia, COPD exacerbations, and asthma showing increased CRP levels due to the inflammatory response in lung tissue (Póvoa, 1998). Similarly, an elevated Erythrocyte sedimentation rate (ESR) is indicative of inflammation, with conditions like tuberculosis and sarcoidosis showing heightened ESR levels (Chopra and Abdel-Naser, 2018). While high ferritin levels are not specific to lung diseases, they can be observed in acute inflammatory states or conditions like hemochromatosis. Rare cases, such as pulmonary hemosiderosis, may also exhibit such levels (Milman and Kirchhoff, 1992). Elevated Lactate dehydrogenase (LDH) can be seen in conditions where there is tissue damage, including pneumonia and pulmonary embolism. LDH might be particularly elevated in conditions like Pneumocystis jirovecii pneumonia, a common condition in immune-compromised individuals (Yale and Limper, 1996). While not directly related to respiratory diseases, elevated BUN and Creatinine levels might indicate kidney dysfunction, which could occur as a secondary effect of conditions like ARDS, affecting multiple organ systems (Bellomo and Ronco, 1998). Lower albumin levels may suggest chronic illness, malnutrition, or liver disease. Some chronic respiratory conditions like COPD can be associated with a decreased albumin level due to chronic inflammation or reduced dietary intake (Schols et al., 1998). Direct correlations between liver enzymes (such as ALT, AST, Bilirubin, GGT) and lung diseases are limited. However, right-sided heart failure induced by severe lung disease (cor pulmonale) can lead to liver congestion and elevated liver enzymes (Alvarez and Mukherjee, 2011). Although not directly linked to respiratory diseases, the total protein test might reflect nutritional status and overall health. Additionally, chronic hypoxia from lung diseases like COPD can stimulate the production of more RBCs, resulting in elevated hemoglobin (HGB) levels (El-Korashy, 2012). Likewise, chronic lung diseases causing hypoxia could lead to an elevated Red blood cell (RBC) count, a condition known as secondary polycythemia (Smith and Landaw, 1978). Elevated White blood count (WBC) could indicate infection or inflammation, often seen in respiratory infections like pneumonia or bronchitis (Macfarlane, 1995).

With the above backdrop, we can observe that blood biomarkers are widely considered as a convenient means to diagnose and assess the severity of respiratory diseases. While many markers show promising results in the early detection of respiratory diseases, it is essential to conduct further large-scale studies to establish their validity and clinical utility. In this pilot study, we retrospectively collected data from a cohort from the local Hospital in Hail, Saudi Arabia. The focus was on the patients who visited the respiratory clinics and were diagnosed with COVID-19, asthma, pneumonia, or other respiratory diseases. Then, statistical analysis and a fitted model were performed to analyze the blood markers that could together indicate these diseases. To the best of our knowledge, this is the first study to combine multiple blood markers and compare them to find possible correlations to the diagnosis of respiratory disease in Hail, Saudi Arabia.

## 2 Materials and methods

### 2.1 Study population

Our study considered a cohort from the local Hospital located in Hail city. The study was approved by the institutional review board (IRB) of Hail University, Kingdom of Saudi Arabia. We considered patients who visited the respiratory clinics in that hospital. Thus, we collected clinical datasets for 913 patients with 1,632 encounters on March 4th, 2025. We did not consider two or more visits within 2 weeks’ time. We did this to avoid the discrepancy and repetitions of the data, and a wider range of different patients with their associated clinical markers.

### 2.2 Study sample

We focused on 15 blood markers to see their contribution to respiratory diseases. We also included the age and gender as different features. We have chosen four different diagnoses’ codes (according to the applied clinical coding). These four diseases represent COVID-19 (U07.1), pneumonia (J18.9, J12.9, and J15.9), asthma (J45.9), and other respiratory complications (U07.2). Regarding the blood markers, we have chosen the C-reactive protein (CRP), Erythrocyte sedimentation rate (ESR), Ferritin, Lactate dehydrogenase (LDH), Blood Urea Nitrogen (BUN), Creatinine, Albumin, ALanine Transaminase (ALT), ASpartate aminoTransferase (AST), Bilirubin, Gamma-Glutamyl Transferase (GGT), total protein, HemoGloBin test (HGB), Red Blood Cell (RBC), and White Blood Count (WBC). The other complications category comprises clinically coded respiratory presentations that did not meet the predefined criteria for COVID-19, pneumonia, or asthma (e.g., non-specific lower/upper respiratory illness or mixed presentations as recorded by the treating team). We recognize that this label is heterogeneous and may affect precision.

### 2.3 Overall statistics

This work is a descriptive/associational analysis using nonparametric tests and multinomial regression for adjusted associations. We did not train or tune predictive models, and we therefore did not perform cross-validation, held-out testing, ROC/PR analysis, or calibration. Where relevant, we report goodness-of-fit diagnostics. We used Python, including NumPy, Pandas packages, to do the analysis and calculate the averages, standard deviations. Also, we used Python to plot figures using the Matplotlib package. In addition, we used model fitness to see if the data we have can be learned to predict the diagnosis or the disease, where we used the Sklearn package in Python to generate a decision tree classifier for 4 different classes. These classes represent the four different diseases.

### 2.4 Setting and period

We retrospectively assembled encounters from 2019 to 2020 dataset.

### 2.5 Inclusion criteria

Encounters with a clinician-assigned diagnosis in four categories: COVID-19 (U07.1), Pneumonia (J18.9; J12.9; J15.9 collapsed to J18.9), Asthma (J45.9), other complications (U07.2).

### 2.6 Exclusion criteria

(i) Missing or non-mappable outcome code, (ii) no laboratory result within the analysis window, (iii) duplicate encounters within 14 days of a prior encounter for the same patient (earliest retained), and (iv) implausible laboratory values or unresolved unit conflicts.

### 2.7 Laboratory timing window

For each encounter, we matched laboratory results via a patient-level nearest-date join within ±14 days of the index encounter date; when multiple results existed in the window, the nearest result was used.

### 2.8 Handling repeated encounters

Because patients may re-present, the primary inferential models use cluster-robust standard errors. As a sensitivity analysis, we repeated the analyses on the index encounter only (earliest per patient).

### 2.9 Missing data

We first excluded encounters with fewer than 19 non-missing fields. For descriptive summaries and correlations, analyses were performed on pairwise complete cases; correlation cells with fewer than 20 co-observations were suppressed and null values removed during the correlation calculation process. For predictive modeling, remaining missing laboratory values were imputed as 0 (no missingness indicators were used). For descriptive summaries and nonparametric comparisons, analyses were performed on pairwise complete cases without imputation. To characterize data availability, we computed the proportion of missing values for each biomarker across the full overall cohort. These results are reported in Supplementary Table S5.

### 2.10 Adjusted associations

For each biomarker, we fit a multivariable multinomial logistic regression with diagnosis (CODE; reference U07.1) as the outcome and the biomarker (z-scored) as the predictor, adjusted for age (per decade) and sex (male vs. female). Results are reported as adjusted odds ratios per 1 SD with 95% confidence intervals. We applied Benjamini–Hochberg FDR across all coefficients (15 biomarkers × 3 non-reference contrasts), reporting q-values and considering q < 0.05 significant.

## 3 Results

### 3.1 Overall description of the data, including age and gender distribution

We have performed several tests and studies on the data collected. There are 913 patients with 1,632 visits on different dates (more than 2 weeks for a single patient). Thus, the data distribution based on the gender and age was performed (Table 1). From this table, it shows that the highest age was 101 years old and the youngest patient was 10 years old. It shows that the males are more than the females overall, in 57% male to 43% female, where the male average age was 51 and the female was 53 (both genders’ average is around 52(±17) years old). Then, the distribution of the ages is based on three different levels, where the first one is between 40 and 60 years old, the second level is below 40 years old, and the last one is above 60 years old. The first level (the second part from Table 1) has around 641 patients (60% male and 40% female) with an average age of around 50 (±6) years old. The second level has 449 patients (57% male and 43% female) with an average age of around 31 (±6) years. The last level has around 532 patients (53% male and 47% female) with an average age of around 72 (±8) years. Overall, based on the analysis of the age and gender, 40% of the patients’ ages range between 40 and 60, 27.5% are less than 40, and lastly, 32.5% are above 60 years old.

**Table 1:** Shows the distribution of the patients based on age and gender.

Gender\age	Counts	Mean (std. dev)	Median	Minimum	Maximum
Age	All ages				
Male	926	51.04 (±17.08)	49.00	10	101
Female	696	52.76 (±17.32)	52.00	16	93
All	1,622	51.75 (±17.20)	50.50	10	101
	Between 40 and 60 years old inclusive				
Male	387	49.14 (±6.03)	48.00	40	60
Female	254	50.43 (±5.70)	50.00	40	60
All	641	49.65 (±5.92)	49.00	40	60
	Less than 40 years old				
Male	257	31.08 (±6.33)	32.00	10.00	39
Female	192	31.32 (±5.10)	32.00	16.00	39
All	449	31.18 (±5.84)	32.00	10.00	39
	Above 60 years old				
Male	282	71.82 (±8.44)	69.00	61.00	101
Female	250	71.58 (±8.26)	70.00	61.00	93
All	532	71.71 (±8.35)	69.00	61.00	101

Across the 15 biomarkers, overall missingness was substantial (mean 73.37%, median 74.69%; range 52.51–86.95%). The highest missingness was observed for GGT (86.95%), Protein (85.85%), and LDH (85.11%), whereas the lowest was for RBC (52.51%), WBC (52.82%), and BUN (59.13%) (see Supplementary Table S5). Given this pattern, inferential analyses were restricted to pairwise complete cases for each comparison, and results should be interpreted with awareness of the varying data availability across biomarkers.

In multivariable analyses (multinomial logistic regression of diagnosis on each biomarker, adjusted for age and sex; effect per 1-SD increase), we tested 45 contrasts (15 biomarkers × 3 non-reference outcomes vs. U07.1/COVID-19) and controlled multiplicity with Benjamini–Hochberg FDR. Two contrasts remained significant after adjustment (*q* < 0.05), both for J18.9 (Pneumonia) vs. U07.1 (COVID-19) and involving RBC and WBC. No adjusted associations met the FDR threshold for J45.9 (Asthma) or U07.2 (Other). Full results are reported in Supplementary Table S6.

### 3.2 Normal ranges for the blood markers and correlations with the respiratory diseases

Table 2 shows the number of patients based on each blood marker because some other patients for one measure are not tested and thus have null values. Those null values are not counted where the counts column represents the number of real values (not null) in each measure. Then, we calculated the median, standard deviation, minimum, and maximum values for each measure among the patients shown in the count column.

**Table 2:** Shows the number of patients, the median, standard deviation, minimum, and maximum values for each measure.

Measure	Counts	Median	Std. Dev.	Min	Max
CRP	390	2.50	3.93	0.13	24.10
ESR	487	52.00	33.48	1.00	150.00
FERRITIN	381	363.10	437.91	4.10	1650.00
LDH	242	275.50	222.91	4.80	1372.00
BUN	658	5.13	10.20	0.98	139.76
CREATININE	479	79.90	45.56	4.92	490.51
Albumin	428	31.00	5.92	16.00	62.10
ALT	411	34.00	38.70	2.25	309.00
AST	412	29.00	40.09	5.00	407.00
Bilirubin	297	7.10	5.82	0.20	44.50
GGT	212	49.00	86.41	4.00	450.00
Protein	229	67.90	7.25	45.90	89.10
HGB	320	13.55	2.25	1.60	18.50
RBC	766	4.86	0.65	2.62	7.43
WBC	761	7.06	4.03	1.03	44.88

Similar to Table 2 and Supplementary Table 1 summarizes patients’ information, but only those diagnosed with asthma with the known code for asthma (J45.9). Low, high, and normal columns represent the number of asthma patients who have lower values than the normal ranges based on each measure, higher values than the normal ranges, or normal within the ranges for each measure, respectively.

Similarly, Supplementary Tables 2–4 have been generated to show the same information as in Supplementary Table 2, but for patients who were diagnosed with COVID-19 disease (U07.1), other respiratory diseases (other than COVID-19 disease) patients (U07.2), and the pneumonia patients (J18.9, J12.9, and J15.9), respectively.

Table 3 shows the normal ranges for the blood markers. Also, we show the correlation ratio between each blood marker and the diagnosis (diseases) since it measures the relationship between the unordered nominal (4 diseases) column and continuous-value features. Here, we have classified the patients into four different diseases (COVID-19, asthma, pneumonia, and other respiratory diseases). The blood markers that have a higher than 20% correlation are ESR, LDH, GGT, total protein, and HGB.

**Table 3:** Shows the normal ranges for each measure taken from the hospital.

Measure	Min	Max	Unit	Corr. Ratio
CRP	0	0.6	Mg/dL	0.199416
ESR	0	15	Mm/Hour	0.21415
FERRITIN	10	291	Ng/ml	0.174845
LDH	100	190	U/L	0.253731
BUN	2.5	6.4	Mmol/L	0.098134
CREATININE	49	115	Umol/L	0.153747
Albumin	34	50	g/L	0.084836
ALT	14	63	U/L	0.168551
AST	15	37	U/L	0.156218
Bilirubin	3	17	umol/L	0.044861
GGT	5	85	U/L	0.268126
Total Protein	64	82	g/L	0.281393
HGB	11.8	17.2	g/dL	0.249529
RBC	3.85	5.75	10^6/uL	0.163705
WBC	3.6	11.4	10^3/uL	0.194183

The Correlation Ratio between each measure and the diseases (asthma, COVID-19, pneumonia, and other respiratory complications).

In the biomarker-by-diagnosis analyses, we summarize, as shown in Table 4, each test with the following fields. N denotes the number of non-missing observations used for that biomarker (after pairwise deletion with 4 classes), and k is the number of distinct diagnosis categories actually present in those observations (up to 4 in our setting; if k < 2, no omnibus test is performed; if k < 4, at least one class had no data for that biomarker). The Kruskal–Wallis statistic (KW_H), reported with degrees of freedom (KW_df = k − 1), quantifies the rank-based separation among groups; its corresponding *p*-value (KW_p) tests the null hypothesis that all group distributions are identical. Because multiple biomarkers are tested, we control the false discovery rate using Benjamini–Hochberg and report the FDR-adjusted q-value (KW_q); results with q < 0.05 are considered statistically significant. To convey magnitude (not just significance), we report epsilon-squared (KW_epsilon_sq) as an effect size for the Kruskal–Wallis test and interpreted as the proportion of variability attributable to between-group differences (rough benchmarks: ≈0.01 small, ≈0.06 medium, ≈0.14 large). Where N is small or k < 4, results are interpreted cautiously and complemented by descriptive summaries; for biomarkers with significant omnibus tests, post-hoc Dunn comparisons (FDR-adjusted) can be provided to identify which pairs of groups differ.

**Table 4:** Shows the Kruskal–Wallis test for the biomarkers and the diseases (asthma, COVID-19, pneumonia, and other respiratory complications).

Measure	*N*	*k*	KW_H	KW_df	KW_p	KW_epsilon_sq	KW_q
AST	414	4	58.21	3	0.000000	0.134656	0.000000
WBC	770	4	35.31	3	0.000000	0.042183	0.000001
Albumin	430	4	34.85	3	0.000000	0.074762	0.000001
GGT	213	4	33.32	3	0.000000	0.145081	0.000001
FERRITIN	381	4	27.99	3	0.000004	0.066298	0.000010
CRP	390	4	27.81	3	0.000004	0.064277	0.000010
ESR	488	4	26.04	3	0.000009	0.047602	0.000020
CREATININE	487	4	25.75	3	0.000011	0.047100	0.000020
Protein	231	4	24.36	3	0.000021	0.094081	0.000035
BUN	667	4	24.00	3	0.000025	0.031681	0.000037
LDH	243	4	22.93	3	0.000042	0.083396	0.000057
HGB	320	4	21.89	3	0.000069	0.059790	0.000086
RBC	775	4	19.85	3	0.000182	0.021861	0.000210
ALT	413	4	18.49	3	0.000349	0.037868	0.000374
Bilirubin	298	4	0.56	3	0.904577	0.000000	0.904577

As in Table 4, for each biomarker we performed an omnibus Kruskal–Wallis test across the four diagnosis groups. To account for testing multiple biomarkers, we controlled the false discovery rate (FDR) using the Benjamini–Hochberg (BH) procedure at *α* = 0.05 across the set of omnibus *p*-values (*m* = 15). We report BH-adjusted *q*-values, with *q* < 0.05 considered statistically significant, alongside the Kruskal–Wallis effect size epsilon-squared (*ε*^2^) to convey magnitude. The interpretation is based on the omnibus tests, *ε*^2^, and per-group descriptive summaries (medians and IQRs).

### 3.3 Distributions of the values of each blood marker compared with the normal ranges

**Figure 1:**
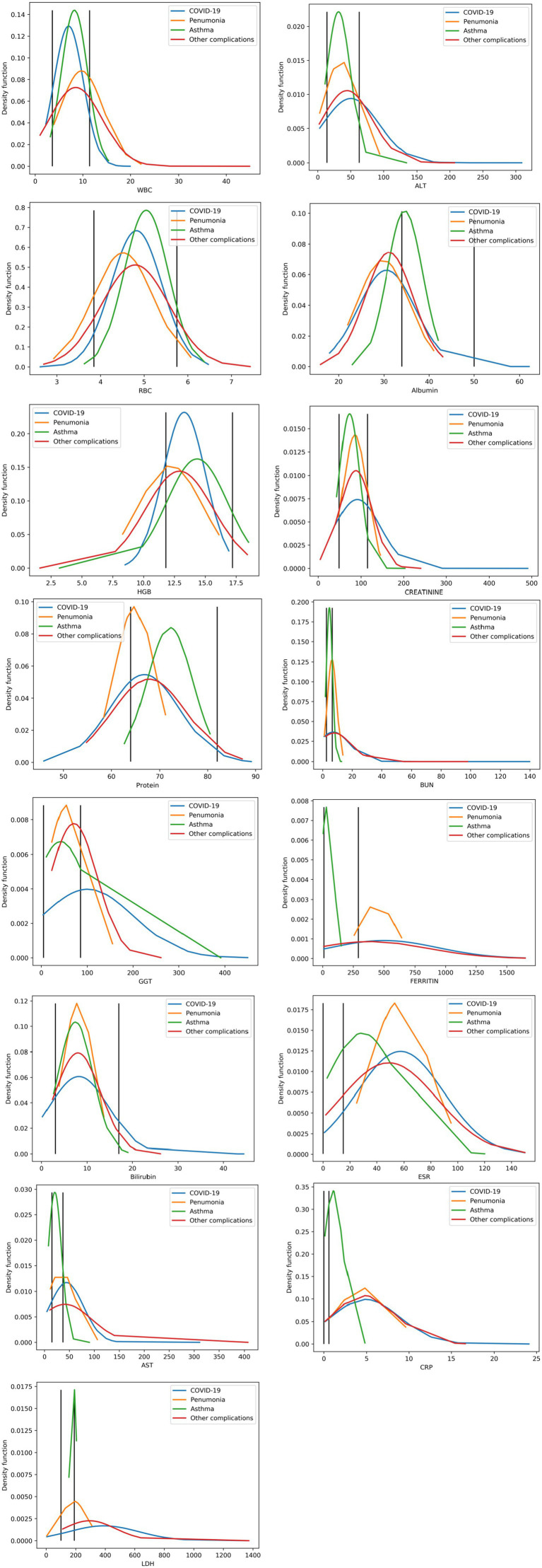
Shows the measures compared to their associated normal ranges. The two vertical black lines represent the minimum and maximum values of the normal ranges.

We have generated a comparison of the normal ranges of each blood marker and COVID-19, pneumonia, asthma, and other respiratory diseases. In each subfigure, black vertical lines represent the minimum and maximum values for normal ranges for the represented blood marker, as shown in Figure 1.

### 3.4 Correlations between the blood markers

**Figure 2:**
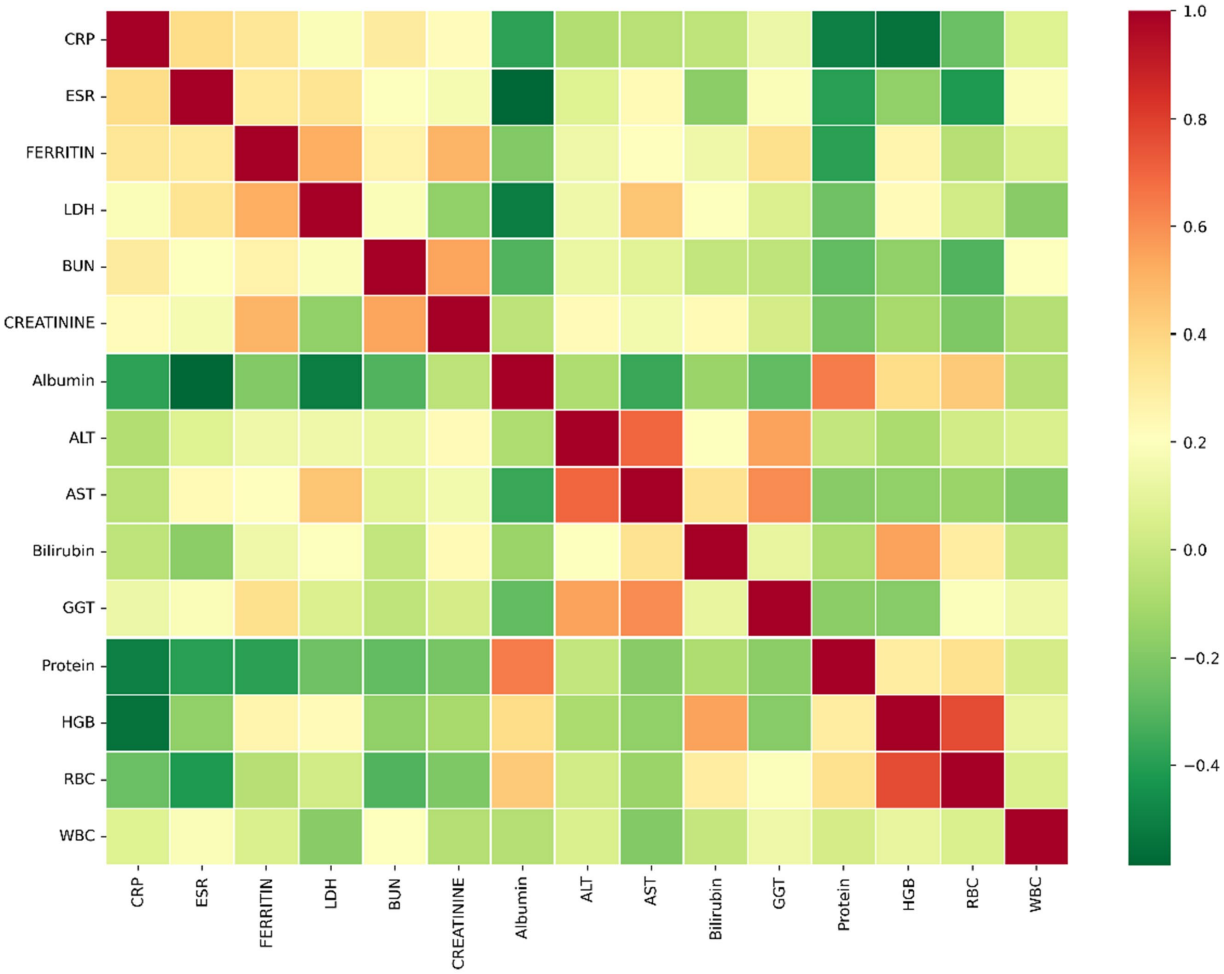
Shows the Spearman correlation coefficients between the two measures (null values removed).

Based on the 15 chosen different blood markers, as there are missing data for patients regarding these markers, we want to highlight this drawback and the lack of information from the hospital. Thus, we generated Table 5, which shows the distribution of the patients based on the available data in each combination of two blood markers. For example, 286 patients have both CRP and ESR values. Our objective from this table is to generate Table 6, which shows the correlation coefficient between each pair of blood markers based on the Spearman correlation coefficient. If the value is positive, it means a positive correlation (when the blood marker increases, the other one tends to increase by a percentage associated with the sign). The same thing is applied when the sign is negative; when blood markers increase, the other one tends to decrease. For example, the correlation coefficient between CRP and HGB (hemoglobin) is 55% negatively correlated. That’s when CRP increases, HGB decreases, and vice versa. Another example of a positive correlation is the correlation between Ferritin and LDH. It is 50% positively correlated. That’s when LDH increases, and Ferritin increases, as well. On the other side, when LDH decreases, Ferritin decreases, as well. Overall, it is noted that the Creatinine and ESR are negatively correlated with RBC. There is also a correlation between GGT and ALT, with a 49% positive correlation. Also, Bilirubin with HGB has a 49% positive correlation, and a 28% positive correlation between HGB with RBC. As shown in Table 6, it is a bit difficult to look at the table with many numbers. Thus, we generated Figure 2, which shows a heat map representing the correlation coefficients as shown in Table 6.

**Table 5:** Shows the number of patients who have laboratory results based on the two measures.

	CRP	ESR	FERRITIN	LDH	BUN	CREATININE	Albumin	ALT	AST	Bilirubin	GGT	Protein	HGB	RBC	WBC
CRP	390	286	149	61	157	87	86	82	78	62	68	53	36	152	154
ESR	286	487	184	72	195	121	117	106	111	83	75	65	68	196	191
FERRITIN	149	184	381	76	131	87	65	60	63	53	50	40	36	121	130
LDH	61	72	76	242	117	65	73	88	77	49	43	43	33	108	109
BUN	157	195	131	117	658	399	201	193	189	117	98	95	125	315	312
CREATININE	87	121	87	65	399	479	130	114	118	74	69	68	134	223	224
Albumin	86	117	65	73	201	130	428	334	341	225	166	196	84	212	210
ALT	82	106	60	88	193	114	334	411	361	223	154	190	69	220	218
AST	78	111	63	77	189	118	341	361	412	225	153	187	75	211	209
Bilirubin	62	83	53	49	117	74	225	223	225	297	143	180	55	146	142
GGT	68	75	50	43	98	69	166	154	153	143	212	158	25	91	95
Protein	53	65	40	43	95	68	196	190	187	180	158	229	25	105	103
HGB	36	68	36	33	125	134	84	69	75	55	25	25	320	317	312
RBC	152	196	121	108	315	223	212	220	211	146	91	105	317	766	737
WBC	154	191	130	109	312	224	210	218	209	142	95	103	312	737	761

**Table 6:** Shows the Spearman correlation coefficients between the two measures (null values removed).

	CRP	ESR	FERRITIN	LDH	BUN	CREATININE	Albumin	ALT	AST	Bilirubin	GGT	Protein	HGB	RBC	WBC
CRP	1.00	0.37	0.33	0.18	0.30	0.22	−0.39	−0.07	−0.05	−0.03	0.13	−0.51	−0.54	−0.26	0.07
ESR	0.37	1.00	0.31	0.34	0.20	0.16	−0.59	0.07	0.23	−0.17	0.18	−0.40	−0.16	−0.42	0.19
FERRITIN	0.33	0.31	1.00	0.52	0.27	0.50	−0.20	0.14	0.21	0.14	0.36	−0.39	0.26	−0.06	0.05
LDH	0.18	0.34	0.52	1.00	0.18	−0.16	−0.52	0.14	0.45	0.20	0.06	−0.24	0.23	0.02	−0.18
BUN	0.30	0.20	0.27	0.18	1.00	0.54	−0.31	0.12	0.09	−0.02	−0.03	−0.28	−0.16	−0.31	0.20
CREATININE	0.22	0.16	0.50	−0.16	0.54	1.00	−0.04	0.23	0.15	0.23	0.03	−0.22	−0.10	−0.21	−0.06
Albumin	−0.39	−0.59	−0.20	−0.52	−0.31	−0.04	1.00	−0.08	−0.36	−0.13	−0.27	0.64	0.37	0.44	−0.06
ALT	−0.07	0.07	0.14	0.14	0.12	0.23	−0.08	1.00	0.70	0.21	0.55	−0.02	−0.09	0.02	0.05
AST	−0.05	0.23	0.21	0.45	0.09	0.15	−0.36	0.70	1.00	0.35	0.61	−0.18	−0.16	−0.13	−0.19
Bilirubin	−0.03	−0.17	0.14	0.20	−0.02	0.23	−0.13	0.21	0.35	1.00	0.11	−0.08	0.55	0.30	−0.01
GGT	0.13	0.18	0.36	0.06	−0.03	0.03	−0.27	0.55	0.61	0.11	1.00	−0.17	−0.19	0.19	0.13
Protein	−0.51	−0.40	−0.39	−0.24	−0.28	−0.22	0.64	−0.02	−0.18	−0.08	−0.17	1.00	0.30	0.35	0.03
HGB	−0.54	−0.16	0.26	0.23	−0.16	−0.10	0.37	−0.09	−0.16	0.55	−0.19	0.30	1.00	0.77	0.11
RBC	−0.26	−0.42	−0.06	0.02	−0.31	−0.21	0.44	0.02	−0.13	0.30	0.19	0.35	0.77	1.00	0.05
WBC	0.07	0.19	0.05	−0.18	0.20	−0.06	−0.06	0.05	−0.19	−0.01	0.13	0.03	0.11	0.05	1.00

### 3.5 Goodness of the fitted model

**Figure 3:**
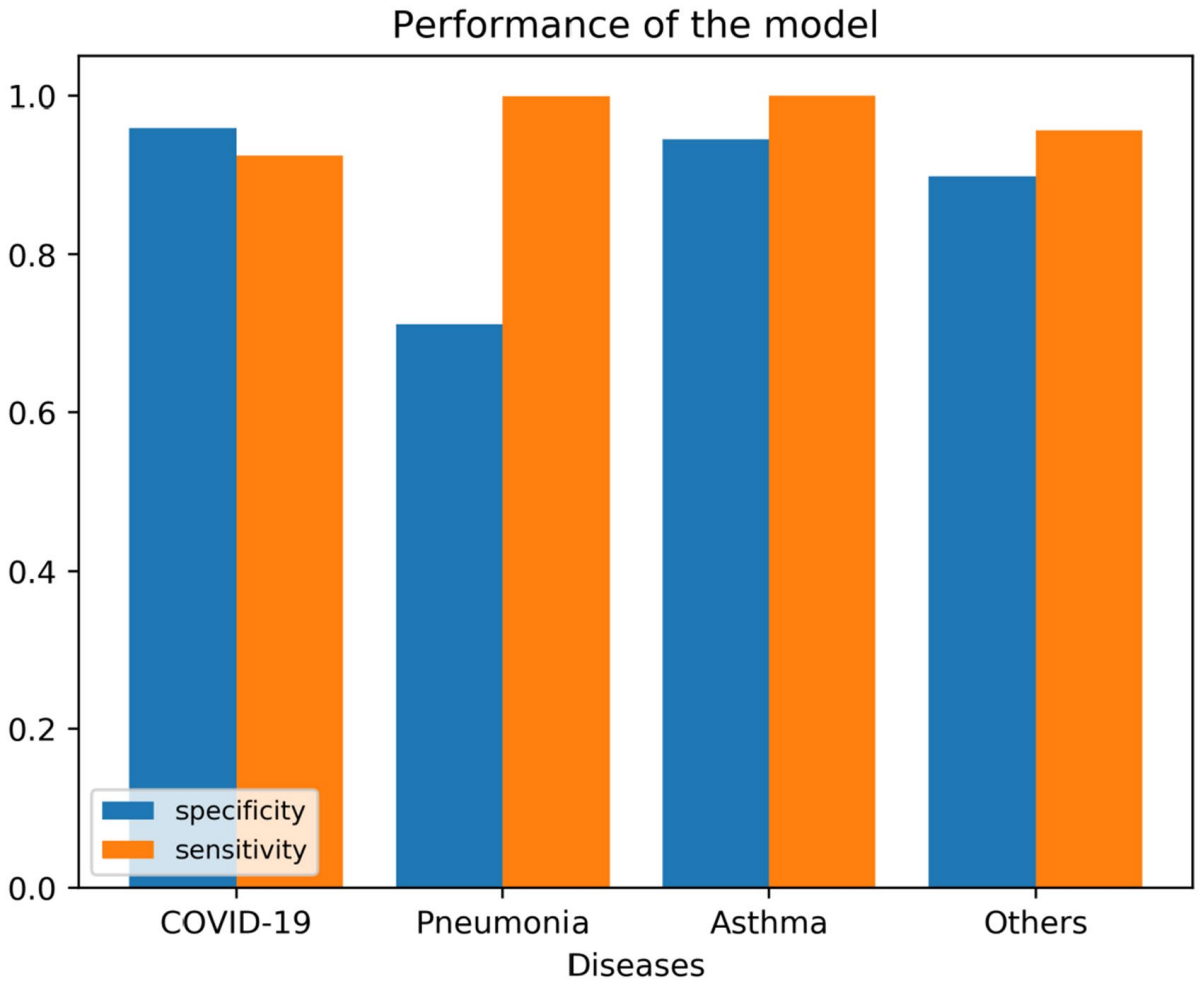
Shows the sensitivity and specificity of the model on the fitted data.

**Figure 4:**
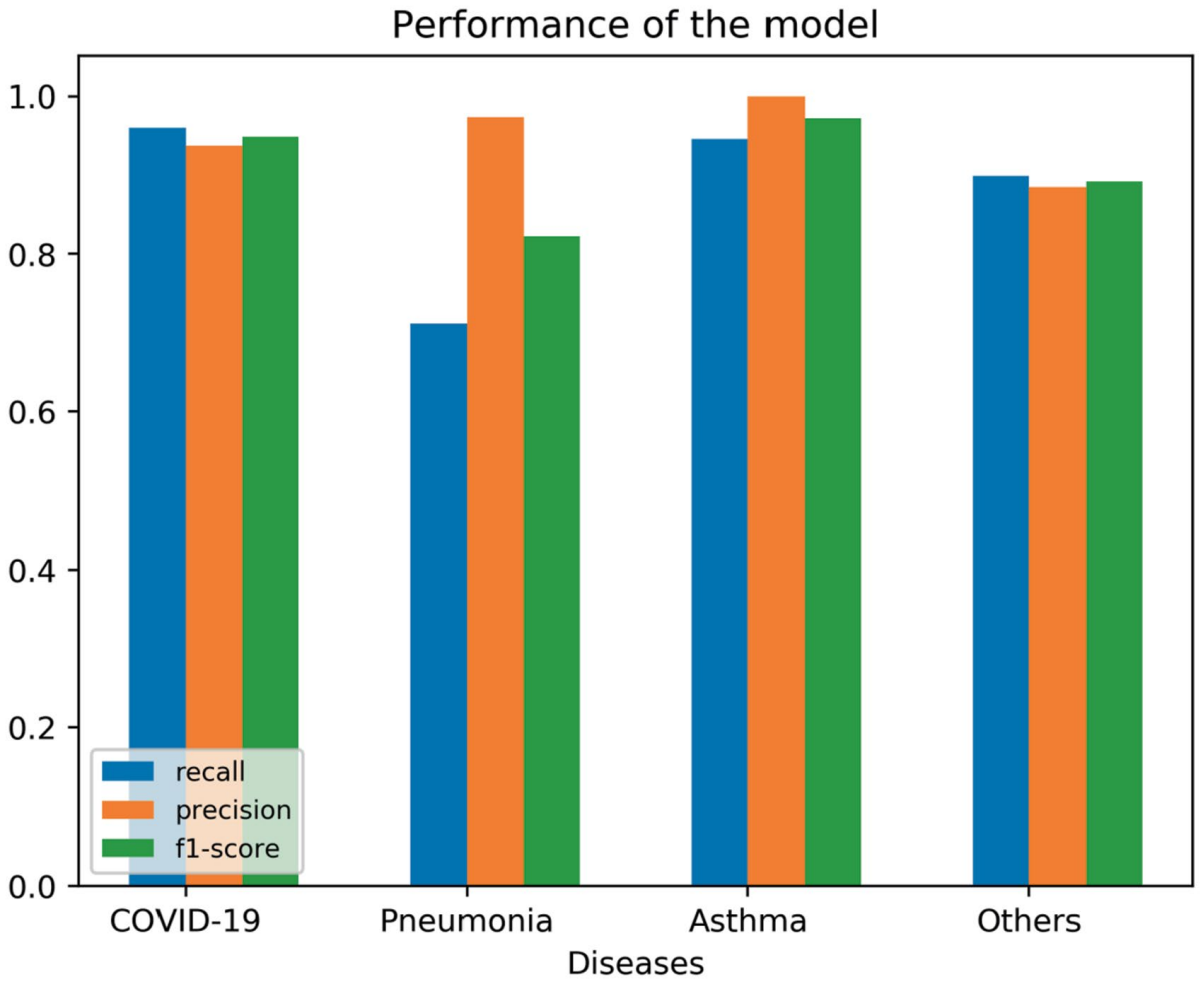
Shows the performance of the goodness of fit for the model based on recall, precision, and F1-score measures.

To see if multiple variables could lead to a specific diagnosis, we used a decision tree algorithm (max tree 30). We had a good fit of the model, as shown in the following tables. Table 7 shows the confusion matrix for the four different diseases, where the rows represent the actual number of patients who are diagnosed with their corresponding diseases. The columns represent the predicted number of patients to be diagnosed in each disease, as shown in each column. Tables 8, 9 show the goodness of the fitted model on the whole dataset based on different metrics, where we used precision, recall, and F1-score. Table 8 evaluates the model without having the age and gender into account; whereas Table 9 takes this into account. To show more details of the model, we generated Figure 3, which shows the performance based on the sensitivity and specificity of the model. Also, Figure 4 is generated to show the performance based on other different measures, which are recall, precision, and F1-score, as shown in the table.

**Table 7:** Shows the confusion matrix for the goodness of fit of the decision tree algorithm.

Actual/predicted	COVID-19	Pneumonia	Asthma	Not COVID-19	Actual total
COVID-19	855	0	0	29	884
Pneumonia	11	41	0	1	53
Asthma	1	1	246	10	258
Not COVID-19	44	1	1	391	437
Predicted total	911	43	247	431	1,632

**Table 8:** Shows the performance of the whole data based on precision, recall, and F1-score measures.

Disease	Precision	recall	f1-score	Support
COVID-19	0.94	0. 97	0.95	884
Pneumonia	0.95	0.77	0.85	53
Asthma	1	0.95	0.97	258
Other complications	0.91	0.89	0.90	437

**Table 9:** Shows the performance of the whole data when including the age and gender information based on precision, recall, and F1-score measures.

Disease	Precision	Recall	f1-score	Support
COVID-19	0.94	0. 98	0.96	884
Pneumonia	0.95	0.79	0.87	53
Asthma	1	0.99	0.99	258
Other complications	0.96	0.89	0.93	437

### 3.6 Age and gender information effects on the fitted model

To assess the contribution of basic demographic factors, we trained two otherwise identical models: a baseline without demographics and a demographically augmented variant that ingests age and gender. Table 8 shows the per-class precision, recall, and F1-scores and the corresponding aggregate metrics.

Overall, incorporating age and gender yields consistent performance gains across diagnostic categories. The demographically augmented model exhibits higher recall for the major disease classes and improved precision where classes are clinically overlapping (e.g., “other complications”), translating into higher overall F1 at both the macro and support-weighted levels. These improvements are observed despite class imbalance, indicating that demographic cues function as informative priors that help the classifier resolve borderline cases without sacrificing specificity.

Table 8 suggests that adding age and gender enhances discrimination in a way that is both practically meaningful and methodologically sound, provided that deployment is accompanied by routine bias monitoring and calibration checks.

## 4 Discussion

This study analyzed routine laboratory biomarkers in four respiratory disease groups and examined their joint behavior and diagnostic relevance. As summarized in Figure 1, several inflammation-linked analytes (e.g., CRP, ESR, LDH) exhibited distributions that are directionally consistent with known pathophysiology, while other markers displayed disease-specific density shifts that help differentiate between entities such as COVID-19, asthma, pneumonia, and other complications. These aggregate patterns support the premise that routinely collected blood tests can carry clinically meaningful signal for triage and decision support.

We further evaluated whether incorporating basic demographic information enhances model behavior. Table 9 contrasts an otherwise identical model trained with versus without age and gender. The demographically augmented specification demonstrates consistently improved discrimination across classes, primarily through gains in recall for the major categories and improved precision where clinical overlap is expected (e.g., “other complications”). Conceptually, these variables act as low-cost priors that help the model resolve borderline cases without materially sacrificing specificity.

At the same time, it is important to contextualize biomarker signal. Several analytes considered here (e.g., liver enzymes, creatinine, CRP) can reflect systemic illness severity rather than pathology confined to the respiratory tract. We have therefore moderated causal language and interpret the associations as indicators of overall physiological stress that, in combination with clinical context, may assist physician judgment.

This work has notable limitations. First, it is a single-center study, which may limit generalizability to settings with different case mix and practice patterns. We suggest combine different datasets to do further analysis (Alghadhban et al., 2025). Second, while age and gender were available and analyzed (Table 9), structured comorbidity fields were not available in the current dataset. Residual confounding is therefore possible, and future data collections should incorporate comorbidity profiles and medication history to enable explicit adjustment and subgroup analyses. Third, although our primary objective was to characterize associations rather than to develop a deployable classifier, we included a comparative model analysis to contextualize effect sizes; rigorous external validation and prospective evaluation remain necessary before any clinical use.

The category labeled “other complications” aggregates clinically coded respiratory presentations that do not meet the criteria for the three primary labels (e.g., non-specific or mixed presentations as recorded by the treating team). We recognize that such heterogeneity can dilute class purity and affect precision/recall; future work should refine this label through stricter coding guidelines or by subdividing it once larger annotated cohorts are available.

In sum, routine laboratory biomarkers, especially when paired with minimal demographics, provide reproducible signals aligned with clinical expectations and can support downstream machine-learning models for respiratory care. To translate these findings, subsequent studies should include comorbidities and medications to reduce confounding, perform subgroup/fairness analyses by age and gender, and conduct external validation and calibration to ensure robustness across populations and platforms. Because our aim was association rather than prediction, we did not include train/test model evaluation; this will be the focus of future work.

## 5 Conclusion

This study underscores the diagnostic potential of blood biomarkers in distinguishing between major respiratory diseases, including COVID-19, pneumonia, asthma, and other complications. The integration of biomarker correlation analysis and machine learning-based classification (decision tree models) demonstrated high diagnostic accuracy, reinforcing the role of non-invasive biomarker-driven diagnostics in clinical practice. Notably, CRP and HGB, Ferritin and LDH, and other biomarker pairs showed statistically significant correlations that align with known inflammatory and metabolic pathways, further validating their role in respiratory disease progression.

The findings of this study provide a novel framework for utilizing biomarker relationships as predictive indicators in respiratory disease classification. Unlike traditional diagnostic approaches, which often rely on imaging and symptomatic assessments, this study presents an AI-driven, cost-effective, and scalable diagnostic strategy. The proposed methodology can be integrated into clinical workflows to enhance early disease detection, optimize patient stratification, and improve treatment decision-making. However, this study has some limitations. The dataset, though extensive, is limited to a single medical center, and external validation in diverse populations and healthcare settings is required. Additionally, while decision tree classifiers provided strong performance, comparative evaluations with deep learning and multi-marker integration models could further enhance predictive accuracy.

Future research should focus on expanding the biomarker panel, incorporating multi-omics data (genomics, proteomics, metabolomics), and exploring real-time biomarker monitoring systems for dynamic disease progression analysis. A translational approach integrating AI-driven biomarker profiling into personalized medicine platforms could revolutionize respiratory disease diagnostics, ultimately improving patient outcomes and reducing healthcare burdens.

## Data Availability

The raw data supporting the conclusions of this article will be made available by the authors, without undue reservation.

## References

[ref1] Alghadhban A. Ramadan R. A. Alazmi M. (2025). Advancing respiratory disease diagnosis: a deep learning and vision transformer-based approach with a novel X-ray dataset. Comput. Biol. Med. 194:110501. doi: 10.1016/j.compbiomed.2025.110501, PMID: 40494170

[ref11] Alvarez A. M. Mukherjee D. (2011). Liver abnormalities in cardiac diseases and heart failure. Int J Angiol. 20, 135–142. 22942628 10.1055/s-0031-1284434 PMC3331650

[ref2] Bellomo R. Ronco C. (1998). Acute renal failure in the intensive care unit: adequacy of dialysis and the case for continuous therapies. Nephrol. Dial. Transplant. 13, 1398–1402. 8671809

[ref23] Blutt S. E. Coarfa C. Neu J. Pammi M. (2023). Multiomic investigations into lung health and disease. Microorganisms. 11:2116. 37630676 10.3390/microorganisms11082116 PMC10459661

[ref3] Cedrés S. Torrejon D. Martínez A. Martinez P. Navarro A. Zamora E. . (2012). Neutrophil to lymphocyte ratio (NLR) as an indicator of poor prognosis in stage IV non-small cell lung cancer. Clin. Transl. Oncol. 14, 864–869. doi: 10.1007/s12094-012-0872-5, PMID: 22855161

[ref4] Chang Y. Wu T. (2022). *Challenges in biomarker-based predictions for lung diseases*. Diagnostic challenges in pulmonology.

[ref5] Chopra A. Abdel-Naser A. (2018). Erythrocyte sedimentation rate. Treasure Island, FL: StatPearls.

[ref6] Christ-Crain M. Müller B. (2005). Procalcitonin in bacterial infections–hype, hope, more or less? Swiss Med. Wkly. 135, 451–460. doi: 10.4414/smw.2005.11169, PMID: 16208582

[ref8] El-Korashy R. I. (2012). Study the relationship of erythropoietin and chronic obstructive pulmonary disease. Egypt J Chest Dis Tuberc. 61, 43–48.

[ref7] Guerra S. Halonen M. Vasquez M. M. Spangenberg A. Stern D. A. Morgan W. J. . (2015). The relation of circulating CC16 to lung function growth, decline, and development of COPD across the lifespan. Lancet Respir Med. 3, 613–620. 26159408 10.1016/S2213-2600(15)00196-4 PMC4640928

[ref17] Hamai K. Iwamoto H. Ishikawa N. Horimasu Y. Masuda T. Miyamoto S. . (2016). Comparative study of circulating MMP-7, CCL18, KL-6, SP-A, and SP-D as disease markers of idiopathic pulmonary fibrosis. Dis Markers. 2016:4759040. 27293304 10.1155/2016/4759040 PMC4886062

[ref10] Larsson K. Janson C. Ställberg B. Lisspers K. Olsson P. Kostikas K. . (2019). Impact of COPD diagnosis timing on clinical and economic outcomes: the ARCTIC observational cohort study. Int J Chron Obstruct Pulmon Dis. 14, 995–1008. 31190785 10.2147/COPD.S195382 PMC6526023

[ref12] Liu X. Cui B. Wang Q. Ma Y. Li L. Chen Z. (2021). Biomarkers for respiratory diseases: present applications and future discoveries. Clin Transl Discov. 1:e11.

[ref13] Macfarlane J. (1995). Prospective study of the incidence, etiology, and outcome of adult lower respiratory tract illness in the community. Thorax 50, 511–518. 11209098 10.1136/thorax.56.2.109 PMC1746009

[ref14] Mannino D. M. Valvi D. Mullerova H. Tal-Singer R. (2012). Fibrinogen, COPD and mortality in a nationally representative U.S. cohort. COPD 9, 359–366. doi: 10.3109/15412555.2012.668249, PMID: 22489912

[ref15] Milman N. Kirchhoff M. (1992). Iron stores in man in relation to diet and iron requirements. Eur. J. Clin. Nutr. 46, 173–185. 9756117 10.1038/sj.ejcn.1600623

[ref25] Okamura K. Takayama K. Izumi M. Harada T. Furuyama K. Nakanishi Y. (2013). Diagnostic value of CEA and CYFRA 21-1 tumor markers in primary lung cancer. Lung Cancer. 80, 45–49. 23352032 10.1016/j.lungcan.2013.01.002

[ref19] Póvoa P. (1998). C-reactive protein: a valuable marker of sepsis. Intensive Care Med. 24, 235–243. doi: 10.1007/s00134-002-1209-6, PMID: 11904651

[ref16] Pujol J. L. Grenier J. Daurès J. P. Daver A. Pujol H. Michel F. B. (1996). CYFRA 21-1 is a prognostic determinant in non-small-cell lung cancer. Br J Cancer. 74, 944–949.

[ref20] Ridker P. M. (2007). C-reactive protein and the prediction of cardiovascular events among those at intermediate risk. J. Am. Coll. Cardiol. 49, 2129–2138. doi: 10.1016/j.jacc.2007.02.052, PMID: 17531663

[ref18] Rosenberg S. R. Kalhan R. (2012). Biomarkers in chronic obstructive pulmonary disease. Transl Res. 159, 228–237. 22424427 10.1016/j.trsl.2012.01.019

[ref28] Saman H. Raza A. Patil K. Uddin S. Crnogorac-Jurcevic T. (2022). Non-invasive biomarkers for early lung cancer detection. Cancers (Basel). 14:5782. 36497263 10.3390/cancers14235782 PMC9739091

[ref22] Schols A. M. Slangen J. Volovics L. Wouters E. F. (1998). Weight loss is a reversible factor in the prognosis of chronic obstructive pulmonary disease. Am. J. Respir. Crit. Care Med. 157, 1791–1797. doi: 10.1164/ajrccm.157.6.9705017, PMID: 9620907

[ref24] Smith J. R. Landaw S. A. (1978). Smokers' polycythemia. N. Engl. J. Med. 298, 6–10. doi: 10.1056/NEJM197801052980102, PMID: 618465

[ref26] Thomsen M. Dahl M. Lange P. Vestbo J. Nordestgaard B. G. (2012). Inflammatory biomarkers and comorbidities in chronic obstructive pulmonary disease. Am J Respir Crit Care Med. 186, 982–988. 22983959 10.1164/rccm.201206-1113OC

[ref27] Wan J. C. M. Massie C. Garcia-Corbacho J. Mouliere F. Brenton J. D. Caldas C. . (2017). Liquid biopsies come of age: towards implementation of circulating tumour DNA. Nat. Rev. Cancer 17, 223–238. doi: 10.1038/nrc.2017.7, PMID: 28233803

[ref21] Wheelock C. E. Goss V. M. Balgoma D. Nicholas B. Brandsma J. Skipp P. J. . (2013). Application of 'omics technologies to biomarker discovery in inflammatory lung diseases. Eur Respir J. 42, 802–825. 23397306 10.1183/09031936.00078812

[ref29] Yale S. H. Limper A. H. (1996). Pneumocystis carinii pneumonia in patients without acquired immunodeficiency syndrome: associated illness and prior corticosteroid therapy. Mayo Clin. Proc. 71, 5–13. doi: 10.4065/71.1.5, PMID: 8538233

[ref9] Yu Y. Z. Gui X. H. Yu M. Huang W. Peng L.-Y. Dai J.-H. . (2022). Soluble ST2 in serum predicts the prognosis of idiopathic pulmonary fibrosis: a retrospective study. Ann Transl Med. 10:797. 35965810 10.21037/atm-22-3215 PMC9372699

