# Supplementary material for: Statistical and machine learning approaches for identifying biomarker associations in respiratory diseases in a population-specific region
Source: Front Artif Intell. 2025 Nov 27;8:1682774. doi: 10.3389/frai.2025.1682774 (PMC12696709; doi:10.3389/frai.2025.1682774)
Supplement: Supplementary file 2 [file Data_Sheet_1.PDF]

| Measure | **counts** | **Median** | **Std. Dev.** | **Min** | **max** | **Low** | **High** | **Normal** |
|---|---|---|---|---|---|---|---|---|
| **CRP** | 18 | 0.85 | 1.20 | 0.13 | 4.80 | 0 | 9 | 9 |
| **ESR** | 29 | 26.00 | 27.69 | 3.00 | 120.00 | 0 | 16 | 13 |
| **FERRITIN** | 9 | 6.80 | 54.67 | 4.10 | 151.40 | 6 | 0 | 3 |
| **LDH** | 3 | 192.00 | 26.50 | 154.00 | 205.00 | 0 | 2 | 1 |
| **BUN** | 84 | 4.24 | 2.08 | 1.77 | 12.70 | 13 | 9 | 62 |
| **CREATININE** | 94 | 68.00 | 24.16 | 43.13 | 202.88 | 12 | 3 | 79 |
| **Albumin** | 57 | 34.00 | 3.96 | 23.00 | 42.00 | 23 | 0 | 34 |
| **ALT** | 59 | 27.00 | 18.15 | 11.00 | 134.00 | 2 | 2 | 55 |
| **AST** | 57 | 17.00 | 13.68 | 8.00 | 90.00 | 15 | 4 | 38 |
| **Bilirubin** | 45 | 6.50 | 3.90 | 2.60 | 19.00 | 2 | 2 | 41 |
| **GGT** | 38 | 28.50 | 60.15 | 11.00 | 391.00 | 0 | 2 | 36 |
| **Protein** | 41 | 72.30 | 4.81 | 62.70 | 80.50 | 1 | 0 | 40 |
| **HGB** | 78 | 14.40 | 2.47 | 3.18 | 18.50 | 11 | 8 | 59 |
| **RBC** | 129 | 5.02 | 0.51 | 3.63 | 6.38 | 1 | 15 | 113 |
| **WBC** | 129 | 7.80 | 2.78 | 3.18 | 15.38 | 4 | 16 | 109 |

**Supplementary Table 1 shows the number of the asthma disease patients (J45.9), the median, standard deviation, minimum and maximum values for each measure. Low, high, and normal counts are the number of the patients based on the normal ranges taken from the hospital.**

| Measure | **Counts** | **Median** | **Std. Dev.** | **Min** | **max** | **Low** | **High** | **Normal** |
|---|---|---|---|---|---|---|---|---|
| **CRP** | 260 | 4.80 | 4.04 | 0.16 | 24.10 | 0 | 237 | 23 |
| **ESR** | 316 | 55.00 | 32.11 | 1.00 | 150.00 | 0 | 279 | 37 |
| **FERRITIN** | 326 | 390.90 | 436.60 | 9.20 | 1650.00 | 1 | 198 | 127 |
| **LDH** | 156 | 311.00 | 237.68 | 4.80 | 1358.00 | 1 | 136 | 19 |
| **BUN** | 378 | 5.30 | 10.92 | 0.98 | 139.76 | 30 | 123 | 225 |
| **CREATININE** | 255 | 84.66 | 53.92 | 40.10 | 490.51 | 14 | 23 | 218 |
| **Albumin** | 228 | 30.00 | 6.36 | 18.00 | 62.10 | 172 | 4 | 52 |
| **ALT** | 211 | 38.00 | 42.56 | 2.95 | 309.00 | 4 | 47 | 160 |
| **AST** | 218 | 35.00 | 34.26 | 5.00 | 311.00 | 17 | 100 | 101 |
| **Bilirubin** | 170 | 7.25 | 6.59 | 0.20 | 44.50 | 13 | 7 | 150 |
| **GGT** | 117 | 58.00 | 100.83 | 4.00 | 450.00 | 2 | 45 | 70 |
| **Protein** | 129 | 66.30 | 7.34 | 45.90 | 89.10 | 49 | 6 | 74 |
| **HGB** | 169 | 13.40 | 1.73 | 8.50 | 16.90 | 32 | 0 | 137 |
| **RBC** | 387 | 4.84 | 0.58 | 2.62 | 6.47 | 14 | 22 | 351 |
| **WBC** | 391 | 6.33 | 3.12 | 2.23 | 19.92 | 29 | 33 | 329 |

**Supplementary Table 2 shows the number of the COVID-19 disease patients (U07.1), the median, standard deviation, minimum and maximum values for each measure. Low, high, and normal counts are the number of the patients based on the normal ranges taken from the hospital.**

| Measure | **Counts** | **Median** | **Std. Dev.** | **Min** | **Max** | **Low** | **High** | **Normal** |
|---|---|---|---|---|---|---|---|---|
| **CRP** | 100 | 2.40 | 3.74 | 0.16 | 16.60 | 0 | 93 | 7 |
| **ESR** | 130 | 40.00 | 36.13 | 3.00 | 150.00 | 0 | 97 | 33 |
| **FERRITIN** | 40 | 177.30 | 468.15 | 9.20 | 1650.00 | 1 | 14 | 25 |
| **LDH** | 76 | 255.00 | 178.88 | 112.00 | 1372.00 | 0 | 59 | 17 |
| **BUN** | 174 | 5.19 | 11.31 | 2.02 | 97.91 | 5 | 55 | 114 |
| **CREATININE** | 114 | 84.33 | 38.08 | 4.92 | 239.99 | 6 | 15 | 93 |
| **Albumin** | 131 | 32.00 | 5.37 | 16.00 | 43.00 | 87 | 0 | 44 |
| **ALT** | 133 | 32.00 | 38.04 | 2.25 | 207.00 | 6 | 23 | 104 |
| **AST** | 127 | 29.00 | 53.59 | 11.00 | 407.00 | 17 | 41 | 69 |
| **Bilirubin** | 75 | 6.20 | 5.10 | 2.36 | 26.10 | 9 | 5 | 61 |
| **GGT** | 51 | 52.00 | 51.82 | 23.00 | 261.00 | 0 | 11 | 40 |
| **Protein** | 51 | 67.20 | 7.82 | 54.80 | 87.20 | 17 | 4 | 30 |
| **HGB** | 62 | 13.05 | 2.79 | 1.60 | 18.40 | 14 | 3 | 45 |
| **RBC** | 228 | 4.84 | 0.78 | 2.70 | 7.43 | 22 | 19 | 187 |
| **WBC** | 219 | 7.51 | 5.52 | 1.03 | 44.88 | 17 | 34 | 168 |

**Supplementary Table 3 shows the number of who have other respiratory diseases (other than the covid-19 disease) patients (U07.2), the median, standard deviation, minimum, and maximum values for each measure. Low, high, and normal counts are the number of the patients based on the normal ranges taken from the hospital.**

| Measure | **counts** | **median** | **Std. Dev.** | **Min** | **Max** | **Low** | **High** | **Normal** |
|---|---|---|---|---|---|---|---|---|
| **CRP** | 12 | 4.80 | 3.36 | 0.60 | 9.60 | 0 | 11 | 1 |
| **ESR** | 12 | 50.00 | 22.42 | 25.00 | 95.00 | 0 | 12 | 0 |
| **FERRITIN** | 6 | 462.50 | 154.37 | 257.50 | 641.10 | 0 | 5 | 1 |
| **LDH** | 7 | 213.00 | 95.74 | 4.80 | 309.00 | 1 | 5 | 1 |
| **BUN** | 22 | 5.42 | 3.26 | 1.08 | 13.62 | 1 | 10 | 11 |
| **CREATININE** | 16 | 84.85 | 28.75 | 47.13 | 145.30 | 1 | 2 | 13 |
| **Albumin** | 12 | 29.00 | 5.98 | 22.00 | 41.00 | 9 | 0 | 3 |
| **ALT** | 8 | 23.00 | 28.50 | 2.95 | 94.00 | 1 | 1 | 6 |
| **AST** | 10 | 20.50 | 30.08 | 12.00 | 106.00 | 2 | 3 | 5 |
| **Bilirubin** | 7 | 7.70 | 3.63 | 3.80 | 13.70 | 0 | 0 | 7 |
| **GGT** | 6 | 39.00 | 49.35 | 23.00 | 155.00 | 0 | 1 | 5 |
| **Protein** | 8 | 64.00 | 4.40 | 58.40 | 71.20 | 4 | 0 | 4 |
| **HGB** | 11 | 12.80 | 2.74 | 8.30 | 16.10 | 4 | 0 | 7 |
| **RBC** | 22 | 4.52 | 0.71 | 2.93 | 6.07 | 4 | 1 | 17 |
| **WBC** | 22 | 9.03 | 4.65 | 3.93 | 22.18 | 0 | 7 | 15 |

**Supplementary Table 4 shows the number of the pneumonia patients (J18.9, J12.9, and J15.9), the median, standard deviation, minimum and maximum values for each measure. Low, high, and normal counts are the number of the patients based on the normal ranges taken from the hospital.**

| | Non-missing counts | Missing counts | Missing percentage |
|---|---|---|---|
| **CRP** | 390 | 1242 | 76.1 |
| **ESR** | 488 | 1144 | 70.1 |
| **FERRITIN** | 381 | 1251 | 76.65 |
| **LDH** | 243 | 1389 | 85.11 |
| **BUN** | 667 | 965 | 59.13 |
| **CREATININE** | 487 | 1145 | 70.16 |
| **Albumin** | 430 | 1202 | 73.65 |
| **ALT** | 413 | 1219 | 74.69 |
| **AST** | 414 | 1218 | 74.63 |
| **Bilirubin** | 298 | 1334 | 81.74 |
| **GGT** | 213 | 1419 | 86.95 |
| **Protein** | 231 | 1401 | 85.85 |
| **HGB** | 320 | 1312 | 80.39 |
| **RBC** | 775 | 857 | 52.51 |
| **WBC** | 770 | 862 | 52.82 |

Supplementary Table 5 shows overall missingness.

| measure | reference disease | compared CODE | Used count | aOR per 1 S.D. | CI95 low | CI95 high | P value | q value |
|---|---|---|---|---|---|---|---|---|
| CRP | U07.1 | J18.9 | 390 | 1.08 | 0.11 | 10.36 | 0.95 | 0.95 |
| CRP | U07.1 | J45.9 | 390 | 0.85 | 0.46 | 1.58 | 0.61 | 0.80 |
| CRP | U07.1 | U07.2 | 390 | 1.31 | 0.89 | 1.92 | 0.17 | 0.40 |
| ESR | U07.1 | J18.9 | 488 | 14.38 | 1.33 | 155.56 | 0.03 | 0.18 |
| ESR | U07.1 | J45.9 | 488 | 0.86 | 0.45 | 1.64 | 0.65 | 0.80 |
| ESR | U07.1 | U07.2 | 488 | 0.89 | 0.61 | 1.30 | 0.55 | 0.80 |
| FERRITIN | U07.1 | J18.9 | 381 | 10.65 | 0.33 | 347.57 | 0.18 | 0.40 |
| FERRITIN | U07.1 | J45.9 | 381 | 0.66 | 0.23 | 1.87 | 0.43 | 0.73 |
| FERRITIN | U07.1 | U07.2 | 381 | 0.80 | 0.45 | 1.41 | 0.44 | 0.73 |
| LDH | U07.1 | J18.9 | 243 | 39.06 | 1.27 | 1203.51 | 0.04 | 0.18 |
| LDH | U07.1 | J45.9 | 243 | 6.24 | 0.76 | 50.99 | 0.09 | 0.32 |
| LDH | U07.1 | U07.2 | 243 | 0.88 | 0.54 | 1.45 | 0.62 | 0.80 |
| BUN | U07.1 | J18.9 | 667 | 10.81 | 2.00 | 58.33 | 0.01 | 0.08 |
| BUN | U07.1 | J45.9 | 667 | 1.26 | 0.54 | 2.92 | 0.60 | 0.80 |
| BUN | U07.1 | U07.2 | 667 | 0.91 | 0.70 | 1.19 | 0.50 | 0.78 |
| CREATININE | U07.1 | J18.9 | 487 | 14.56 | 1.75 | 120.83 | 0.01 | 0.10 |
| CREATININE | U07.1 | J45.9 | 487 | 1.05 | 0.32 | 3.48 | 0.93 | 0.95 |
| CREATININE | U07.1 | U07.2 | 487 | 0.83 | 0.60 | 1.15 | 0.27 | 0.52 |
| Albumin | U07.1 | J18.9 | 430 | 1.26 | 0.13 | 12.51 | 0.84 | 0.93 |

| | | | | | | | | |
|---|---|---|---|---|---|---|---|---|
| Albumin | U07.1 | J45.9 | 430 | 12.21 | 0.63 | 237.84 | 0.10 | 0.32 |
| Albumin | U07.1 | U07.2 | 430 | 1.42 | 0.95 | 2.12 | 0.09 | 0.32 |
| ALT | U07.1 | J18.9 | 413 | 6.16 | 0.45 | 84.59 | 0.17 | 0.40 |
| ALT | U07.1 | J45.9 | 413 | 1.42 | 0.40 | 5.08 | 0.59 | 0.80 |
| ALT | U07.1 | U07.2 | 413 | 1.14 | 0.74 | 1.75 | 0.55 | 0.80 |
| AST | U07.1 | J18.9 | 414 | 7.64 | 0.66 | 88.25 | 0.10 | 0.32 |
| AST | U07.1 | J45.9 | 414 | 1.44 | 0.29 | 7.02 | 0.65 | 0.80 |
| AST | U07.1 | U07.2 | 414 | 1.05 | 0.71 | 1.55 | 0.80 | 0.93 |
| Bilirubin | U07.1 | J18.9 | 298 | 85.82 | 2.87 | 2561.92 | 0.01 | 0.10 |
| Bilirubin | U07.1 | J45.9 | 298 | 1.06 | 0.42 | 2.66 | 0.90 | 0.94 |
| Bilirubin | U07.1 | U07.2 | 298 | 0.71 | 0.42 | 1.18 | 0.19 | 0.40 |
| GGT | U07.1 | J18.9 | 213 | 71.67 | 0.93 | 5552.12 | 0.05 | 0.24 |
| GGT | U07.1 | J45.9 | 213 | 1.18 | 0.23 | 6.12 | 0.85 | 0.93 |
| GGT | U07.1 | U07.2 | 213 | 0.65 | 0.36 | 1.18 | 0.16 | 0.40 |
| Protein | U07.1 | J18.9 | 231 | 4.86 | 0.19 | 122.27 | 0.34 | 0.61 |
| Protein | U07.1 | J45.9 | 231 | 1.68 | 0.73 | 3.90 | 0.22 | 0.46 |
| Protein | U07.1 | U07.2 | 231 | 0.94 | 0.58 | 1.54 | 0.82 | 0.93 |
| HGB | U07.1 | J18.9 | 320 | 4.47 | 0.48 | 41.71 | 0.19 | 0.40 |
| HGB | U07.1 | J45.9 | 320 | 1.24 | 0.67 | 2.27 | 0.50 | 0.78 |
| HGB | U07.1 | U07.2 | 320 | 1.02 | 0.73 | 1.45 | 0.89 | 0.94 |
| RBC | U07.1 | J18.9 | 775 | 44.19 | 8.31 | 234.99 | 0.00 | 0.00 |
| RBC | U07.1 | J45.9 | 775 | 0.49 | 0.28 | 0.86 | 0.01 | 0.10 |
| RBC | U07.1 | U07.2 | 775 | 0.76 | 0.59 | 0.98 | 0.03 | 0.18 |
| WBC | U07.1 | J18.9 | 770 | 29.12 | 5.56 | 152.45 | 0.00 | 0.00 |
| WBC | U07.1 | J45.9 | 770 | 0.86 | 0.65 | 1.14 | 0.29 | 0.55 |
| WBC | U07.1 | U07.2 | 770 | 0.81 | 0.63 | 1.05 | 0.11 | 0.32 |
